# WW45, a Gli1 binding protein, negatively regulated Hedgehog signaling in lung cancer

**DOI:** 10.18632/oncotarget.12155

**Published:** 2016-09-21

**Authors:** Xuebing Li, Xuexia Zhou, Yaguang Fan, Yalong Zhang, Lingling Zu, Feng Yao, Qinghua Zhou

**Affiliations:** ^1^ Tianjin Key Laboratory of Lung Cancer Metastasis and Tumor Environment, Tianjin Lung Cancer Institute, Tianjin Medical University General Hospital, Tianjin 300052, China; ^2^ Department of Neuropathology, Tianjin Key Laboratory of Injuries, Variations and Regeneration of the Nervous System, Tianjin Neurological Institute, Tianjin Medical University General Hospital, Tianjin 300052, China; ^3^ Department of Thoracic Surgery, Shanghai Chest Hospital, Shanghai Jiao Tong University, Shanghai 200030, China; ^4^ Sichuan Lung Cancer Institute, Sichuan Lung Cancer Center, West China Hospital, Sichuan University, Chengdu 610041, China

**Keywords:** WW45, lung cancer, Hedgehog signaling, cell growth, migration

## Abstract

Over-expression of Gli1 is very common in lung cancer. However, the underlying molecular mechanism remains largely unknown. Here, using mass spectrum, we have identified WW45 as a binding partner of Gli1. WW45 interacted with Gli1, promoted its ubiquitination and inhibited the expression of its target genes. In the functional studies, WW45 inhibited the growth and migration of lung cancer cells. Knocking down the expression of WW45 promoted the growth and migration of lung cancer cells, which was rescued by down-regulation of Gli1. Moreover, over-expression of WW45 inhibited the tumorigenesis in a *de novo* lung cancer tumorigenesis mouse model (LKB-Ras) as well as the expression of Gli1. Also over-expression of WW45 improved the survival of these mice. In addition, the expression of WW45 was down-regulated in the clinical lung cancer samples, which was inversely correlated with the expression of Gli1. Taken together, this study demonstrated the suppressive roles of WW45 in lung cancer by inhibiting the Hedgehog/Gli1 signaling.

## INTRODUCTION

Lung cancer is one of the leading causes of cancer-related death in the world [1]. Approximately 80% of lung cancer patients suffer from non-small cell lung cancer (NSCLC), which includes lung adenocarcinoma, lung squamous cell carcinoma and lung large cell carcinoma. Due to the lack of detectable initial symptoms and effective treatment options, the 5-year survival rates remain unsatisfactory for most patients, left the median survival for advanced lung cancer patients being 9-12 months [2, 3]. Recently, molecular targeting therapy has shed much light on the therapy of lung cancer [4, 5]. Therefore, comprehensive investigations of differential gene expression as well as the internal functions and mechanisms will promisingly benefit for identifying reliable earlier markers and therapeutic targets for lung cancer.

WW45 is a core component of conserved Hippo (mammalian sterile 20-like kinase, Mst1 and Mst2) signaling [6], which controls the organ size and suppresses the tumorigensis. Activation of Hippo signaling leads to the recruitment of WW45 by Mst1 and Mst2, which apparently down-regulate cyclin E, Drosophila inhibitor of apoptosis protein 1 (DIAP1) and other proteins, and eventually restricts cell cycle progression and promotes apoptosis [7]. WW45 has been demonstrated as a tumor suppressor in liver cancer, by restraining hepatic oval cell proliferation, liver size and liver tumorigenesis collaborated with Hippo signaling [8]. However, the expression pattern and the functions of WW45 in lung cancer remain unclear. Also, although lots of studies have established WW45 as an important regulator for Hippo signaling, the interaction between WW45 and RASSF1 has demonstrated the Hippo pathway-independent functions of WW45 [9]. Further exploring the Hippo pathway-independent functions of WW45 might benefit the cancer treatment in future.

Hedgehog/Gli1 signaling has been reported to be dysregulated in various cancer types, including lung cancer [10]. Activation of Hedgehog/Gli1 signaling promotes the growth, migration, invasion and drug resistance of the lung cancer cells [11]. The binding of Hedgehog ligand to the receptor Patched1 (Ptc1) activates smoothened (Smo) and the transcriptional activity of Gli1, which up-regulates the expression of a panel of target genes, including Patched1 and FOXM1 [10, 12]. The increase of Gli1 protein level and Gli1 nuclear localization are very common in the lung cancer tissues, suggesting Gli1 might be a promising target for lung cancer treatment [10]. However, the regulation of Gli1 degradation is not fully understood. Here, we examined the expression pattern and the functions of WW45 in lung cancer, and further investigated the regulation of Hedgehog signaling by WW45.

## RESULTS

### WW45 interacted with Gli1

**Figure 1:**
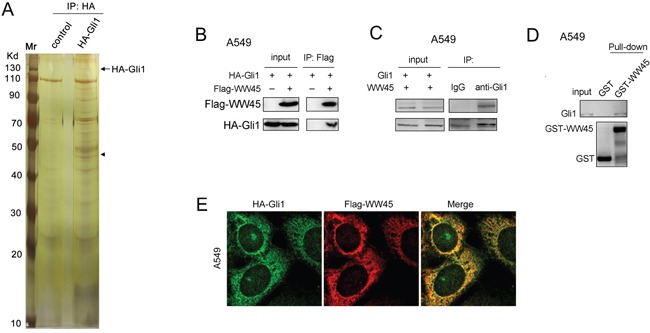
WW45 interacted with Gli1 **A.** Identifying the binding protein of Gli1. The HA-Gli1 plasmid was transfected into HEK293T cells. The immunoprecipitation was performed. The binding protein of Gli1 was separated by SDS-PAGE and identified by mass spectrum. **B.** Ectopically expressed WW45 (Flag-WW45) and Gli1 (HA-Gli1) interacted with each other in A549 cells. **C.** Endogenous WW45 and Gli1 formed a complex in A549 cells. **D.** GST pull-down assay demonstrated that Gli1 bind to GST-WW45. **E.** The immunofluorescence staining assay demonstrated the co-localization of Flag-WW45 and HA-Gli1. Green, HA-Gli1; Red, Flag-WW45. Details about immunofluorescence staining were described in the part of “Materials and Methods”.

To identify the binding partner of Gli1, we first immunoprecipitated the HA-tagged Gli1 and found out the binding protein using mass spectrum (Figure 1A). The results revealed that WW45 was a potential binding protein of Gli1. Next, we examined the interaction between WW45 and Gli1 by immunoprecipitation, immunofluorescence and GST pull-down. Exogenously expressed WW45 (Flag-WW45) and Gli1 (HA-Gli1) were found to interact with each other (Figure 1B). Moreover, endogenously expressed WW45 bound Gli1 (Figure 1C). In addition, in the GST pull-down assay, the fusion protein GST-WW45 was shown to interact with endogenous Gli1 (Figure 1D). Furthermore, the subcellullar localization analysis using immunofluorescence staining demonstrated the co-localization between WW45 and Gli1 (Figure 1E). These results confirmed that WW45 was a binding protein of Gli1.

### WW45 inhibited Hedgehog signaling by promoting the degradation of Gli1

**Figure 2:**
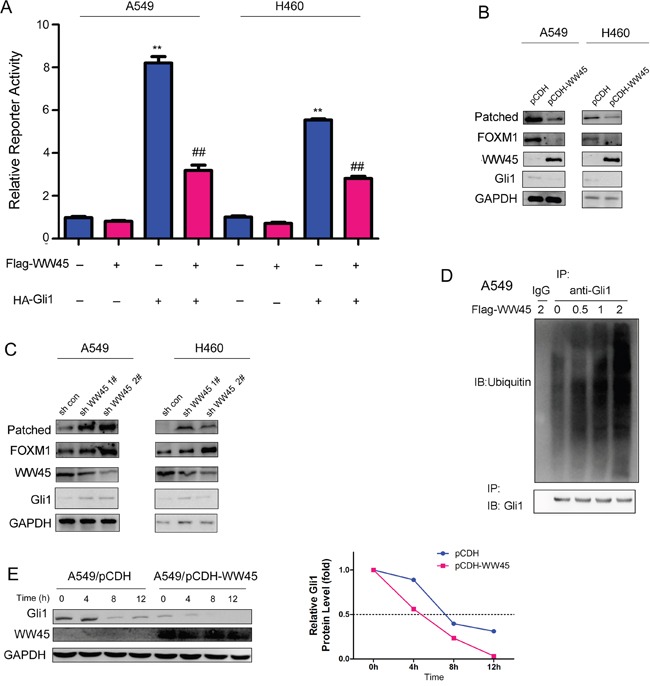
WW45 inhibited the activation of Hedgehog signaling **A.** Luciferase reporter assay demonstrated that WW45 impaired the activation of Hedgehog/Gli1 signaling. **B.** Over-expression of WW45 down-regulated the expression of Hedgehog/Gli1 target genes, such as Patched and FOXM1 in A549 and H460 cells. **C.** Knocking down the expression of WW45 up-regulated the expression of Hedgehog/Gli1 target genes, such as Patched and FOXM1 in A549 and H460 cells. **: *P*<0.01. **D.** WW45 promoted the ubiquitination of Gli1 in a dose-dependent manner. The WW45 expression plasmid (Flag-WW45) was transfected into A549 cells. 48 hours later, cells were treated with MG132. 8 hours later, cells were lysed and the immunoprecipitation was performed using anti-Gli1 antibody. The ubiquitination was examined using the anti-ubiquitin antibody. **E.** The half-life of WW45 was shortened upon the over-expression of WW45. A549 cells were treated with CHX (1μg/ml) and harvested at different time point, and the expression of Gli1 was examined.

The interaction between WW45 and Gli1 prompted us to examine whether WW45 regulated the activity of Hedgehog/Gli1 signaling. It was found that WW45 impaired the activation of Hedgehog signaling induced by Gli1 (Figure 2A), suggesting WW45 as a negative regulator of Hedgehog signaling. In addition, over-expression of WW45 inhibited the expression of Gli1 and its target genes downstream of Gli1, such as Patched and FOXM1, while knocking down the expression of WW45 up-regulated the expression of Gli1, Patched and FOXM1 (Figure 2B–2C). Moreover, in the ubiquitination assay, over-expression of WW45 promoted the ubiquitination of Gli1 in a dose-dependent manner (Figure 2D), suggesting that WW45 inhibited Hedgehog signaling by promoting the degradation of Gli1. Furthermore, we evaluated the effects of WW45 expression on the half-life of Gli1 upon the treatment of CHX (1μg/ml). It was found that the half-life of Gli1 was shortened after the over-expression of WW45 (Figure 2E). Taken together, these findings suggested that WW45 inhibited Hedgehog signaling by promoting the degradation of Gli1.

### WW45 inhibited the growth and migration of lung cancer cells through Hedgehog/Gli1 signaling

**Figure 3:**
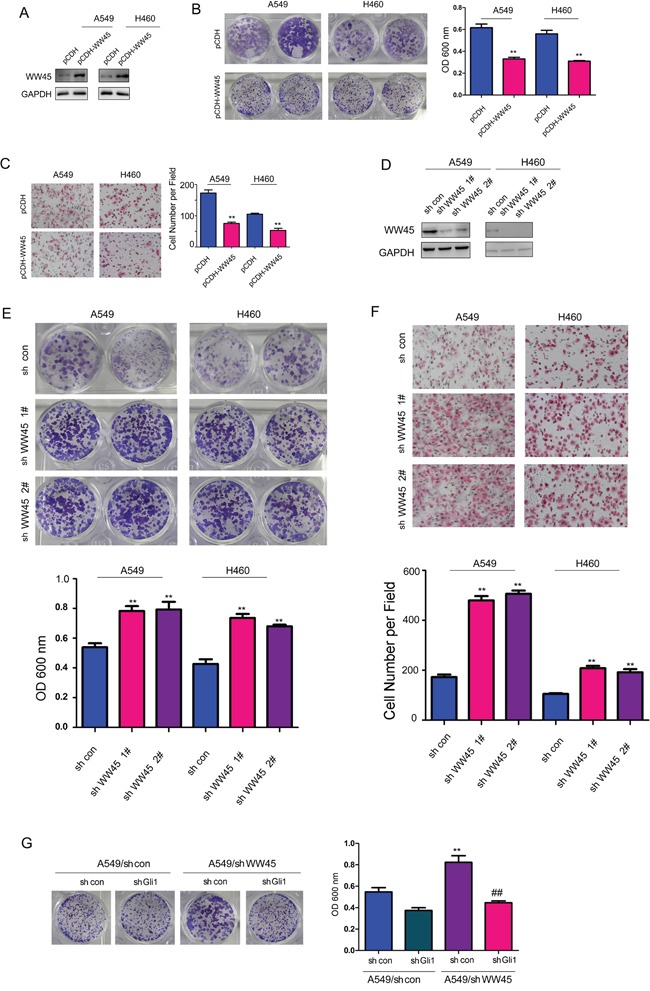
WW45 inhibited the growth and migration of the lung cancer cells through negatively regulating Hedgehog/Gli1 signaling **A.** Over-expression of WW45 in A549 and H460 cells confirmed by western blot. **B.** Crystal violet staining assay demonstrated that WW45 inhibited the growth of A549 and H460 cells. **C.** Cell migration assay using a Boyden Chamber demonstrated that WW45 inhibited the migration of A549 and H460 cells. **: *P*<0.01. **D.** Knocking down the expression of WW45 in A549 and H460 cells confirmed by western blot. **E.** Crystal violet staining assay demonstrated that down-regulation of WW45 promoted the growth of A549 and H460 cells. **F.** Cell migration assay using a Boyden Chamber demonstrated that down-regulation of WW45 promoted the migration of A549 and H460 cells. **: *P*<0.01. **G.** Knocking down the expression of Gli1 rescued the promoting effects of WW45 down-regulation on cell growth. **: *P*<0.01, ##: *P*<0.01.

To investigate the cellular functions of WW45 in lung cancer, we generated stable A549 and H460 cell lines expressing exogenous WW45 (pCDH-WW45) or empty vector (pCDH), which was confirmed by western blot analysis (Figure 3A). Using crystal violet assay, we found that over-expression of WW45 inhibited the growth of A549 and H460 cells (Figure 3B). In addition, the forced expression of WW45 in A549 and H460 cells impaired the migration of these cells (Figure 3C). These observations indicated that up-regulation of WW45 attenuated the tumorigenicity of lung cancer cells, at least by limiting cell growth and migration.

To further study the functional roles of endogenous WW45 in lung cancer, we directly silenced the expression of WW45 by using two lentiviral vectors containing different shRNA that specifically targeting WW45 (Figure 3D). Consistently, knocking down the expression of endogenous WW45 promoted both the growth and migration of A549 and H460 cells, respectively (Figure 3E–3F). Moreover, knocking down the expression of Gli1 abolished the promoting effects on cell growth induced by knocking down the expression of WW45 (Figure 3G). Taken together, these data suggested the tumor suppressive roles of WW45 in lung cancer by inhibiting Hedgehog/Gli1 signaling.

### Over-expression of WW45 inhibited *de novo* lung cancer progression elicited by LKB loss

**Figure 4:**
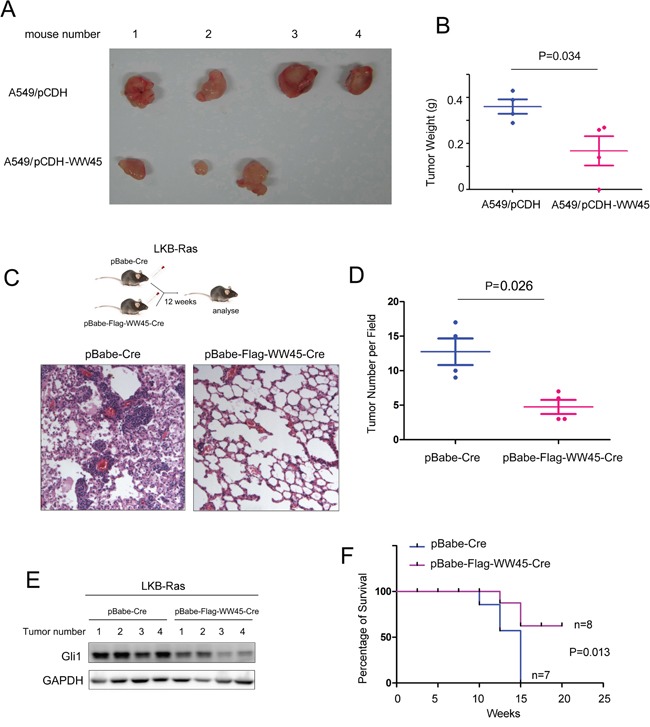
WW45 impaired the tumorigenesis of lung cancer through negatively regulating Hedgehog/Gli1 signaling **A.** Over-expression of WW45 inhibited the tumorigenecity of A549 cells in the nude mice. **B.** The weight of the tumors shown in (A). **C.** Over-expression of WW45 impaired the *de novo* tumorigenesis of lung cancer driven by loss of *LKB1* and mutated *Kras* (*KrasG12D*). The lung tissues were subjected to HE staining. **D.** The number of the tumors shown in (C) was quantified. **E.** The expression of Gli1 in tumors. **F.** Over-expression of WW45 improved the survival of LKB-Ras mice demonstrated by the Kaplan-Meier analysis.*: *P*<0.05.

Next, we over-expressed WW45 in A549 cells and investigated whether WW45 impaired the tumorigenecity of lung cancer cells *in vivo* using the nude mice. The results came out that over-expression of WW45 inhibited the tumorigenesis of A549 in the nude mice (Figure 4A–4B). Moreover, to further investigate whether WW45 suppressed the progression of lung cancer, we used a well-characterized NSCLC mouse model initiated by Kras G12D and LKB1 loss (LKB-Ras in short). LKB-Ras mice were treated with retro-virus expressing Cre and WW45 (Flag-WW45), and analyzed 12 weeks after virus infection. Up-regulation of WW45 reduced the tumor number significantly compared with the control group (Figure 4C–4D). In addition, the expression of Gli1 in the tumors over-expressing WW45 was down-regulated (Figure 4E). Moreover, over-expression of WW45 prolonged the survival of LKB-Ras mice (Figure 4F). Taken together, over-expression of WW45 inhibited the initiation and progression of lung cancer.

### WW45 was down-regulated in clinical lung cancer samples and its expression inversely correlated with Gli1

**Figure 5:**
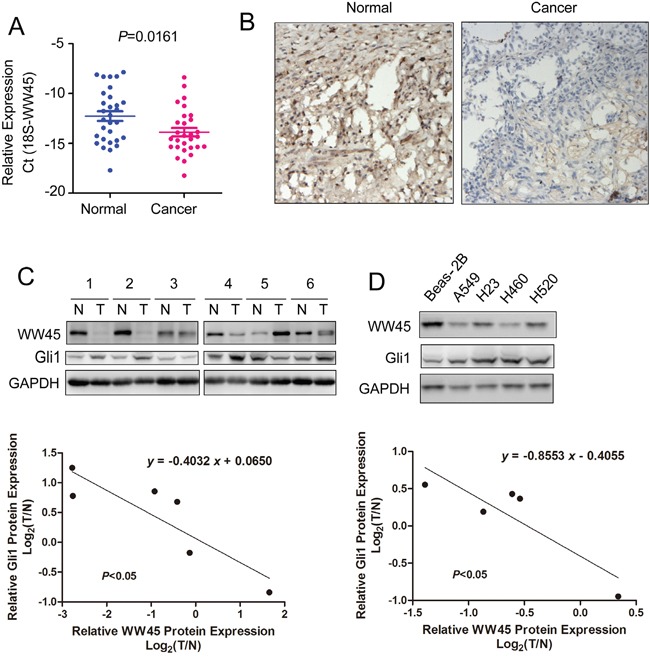
The mRNA and protein levels of WW45 were decreased in lung cancer **A.** The mRNA level of WW45 was decreased in lung cancerous tissues. Expression levels of WW45 in 30 lung cancer samples and adjacent normal specimens were analyzed by qRT-PCR and normalized to the levels of 18S. **B.** The protein level of WW45 was down-regulated in lung cancerous tissues examined by immunohistochemistry. **C.** The expression of WW45 was inversely correlated with Gli1 in lung cancer tissues. The expression of WW45 and Gli1 was examined in six pairs of lung cancer tissues and paired non-cancerous tissues. The correlation between the expression of Gli1 and WW45 was analyzed. **D.** The expression of WW45 and Gli1 in the normal cell line Beas-2B and lung cancer cell lines. The correlation between the expressions of Gli1 and WW45 was analyzed.

To evaluate the potential roles of WW45 in the tumorigenesis of lung cancer, its expression in clinical lung cancer tissues and cell lines were examined using quantitative real-time PCR, western blot analysis and immunohistochemistry. As shown in Figure 5A, the mRNA level of WW45 was significantly decreased in lung cancer tissues compared with the paired non-cancerous tissues (Figure 5A, *P*<0.05). Also, the protein expression of WW45 was down-regulated in cancerous tissues assessed by immunohistochemistry and western blot analysis (Figure 5B–5C). Moreover, WW45 expression was decreased in four lung cancer cell lines (A549, H23, H460 and H520) when compared to the normal human bronchial epithelial cell line, Beas-2B (Figure 5D). Most importantly, the expression of WW45 was inversely correlated with the expression of Gli1 (Figure 5C–5D). These data further demonstrated that down-regulation of WW45 promoted the carcinogenesis of lung cancer by activating Hedgehog signaling.

## DISCUSSION

WW45 was initially identified as a binding partner of mammalian sterile 20-like kinase 1 (Mst1), and promoted the Mst1-mediated apoptosis [13]. Salvador, the homolog of human WW45 in Drosophila, has also been reported to promote both cell cycle exit and apoptosis [14]. These studies confirmed the negative roles of WW45 in the Hippo/YAP signaling. However, whether WW45 was involved in the regulation of other signal pathways remains largely unknown. In this study, we have found that WW45 was dramatically down-regulated in clinical lung cancer tissues and established cell lines, and WW45 inhibited the activation of Hedgehog/Gli1 signaling by promoting the ubiquitination of Gli1. Most importantly, over-expression of WW45 not only inhibited the tumorigenecity of lung cancer cells, but also impaired the *de novo* tumorigenesis of lung cancer driven by loss of LKB1 and mutated Kras (Kras G12D). This study revealed the novel mechanism for the suppressive roles of WW45 in the cancer progression by negatively regulating Hedgehog/Gli1 signaling.

Hedgehog/Gli1 signaling promotes the growth, migration and metastasis of lung cancer [15, 16]. Cyclopamine, the inhibitor for Smo, has shown anti-cancer activity [17]. In this study, we found that over-expression of WW45 promoted the ubiquitination and degradation of Gli1. Previous studies have shown beta-Trcp was an E3 ligase of Gli1 [18]. Although we did not examine the interaction between beta-Trcp and WW45, it is possible that WW45 might promote the ubiquitination of Gli1 by enhancing the interaction between Gli1 and beta-Trcp.

Activation of Hippo/YAP signaling and Hedgehog signaling is very common in the progression of lung cancer [19, 20]. Simultaneously targeting Hippo/YAP signaling and Hedgehog signaling might be a promising strategy for the treatment of lung cancer. Our study combined with the previous reports suggested that WW45 was a common negative regulator for Hippo/YAP signaling and Hedgehog signaling. Therefore, enhancing the function of WW45 might be helpful for the cancer therapy. Although our data are very promising, further study using the WW45 knocking out mice will provide more insights into its functions.

## MATERIALS AND METHODS

### Patients and tissues

A total of 30 lung cancer tissues and paired non-cancerous tissues were obtained from patients who received surgery at Shanghai Chest Hospital, Shanghai Jiao Tong University. The written informed consent was obtained from the patients. This study was approved by the ethics committee of the hospital. The diagnosis of lung cancer was histologically confirmed and none of the patients received preoperative therapy. All of the patients agreed with this study. Cancer tissues and paired non-cancerous tissues were stored at −80°C in a freezer.

### Cell lines and cell culture

Lung cancer cell lines (A549, H460, H23 and H520) and normal human bronchial epithelial cell line (Beas-2B) were obtained from Cell Line Resource Center, Shanghai Institute of Biochemistry and Cell Biology, the Chinese Academy of Sciences (Shanghai, China). All cells were cultured in RPMI-1640 medium supplemented with 10% fetal bovine serum (GIBCO), 100 units/mL penicillin, and 100μg/mL streptomycin in water-warmed incubator with 5% CO_2_ at 37°C.

### Quantitative real-time PCR

Total RNA was extracted using TRIzol. The complementary DNA (cDNA) was prepared as previously described [8]. The expression of WW45 in the lung tissues was examined by quantitative real-time PCR using SYBR® Green Realtime PCR Master Mix (TOYOBO) following the instructions of the manufacturer. Sequences of quantitative real-time PCR primers are listed as follows:

18S Forward primer: 5′-TAAATCAGTTATGGTTCCTT-3′

18S Reverse primer: 5′-CGACTACCATCGAAAGTTGA-3′

WW45 Forward primer: 5′-ATCTTATGCCTTCATTCATC-3′

WW45 Reverse primer: 5′-AGATAAGAAGGTGCAGA TAAT-3′

### Immunohistochemistry

Immunohistochemistry was performed as previously described [8]. Briefly, paraffin-embedded lung cancer tissues and paired non-cancerous tissues were subjected to deparaffinization, antigen recovery and incubated with the anti-WW45 antibody (Cell Signaling Technology; 1:100) for following DAB horseradish peroxidase color development.

### Western blot

Western blot analysis was performed as previously described [8]. Cells were lysed in RIPA lysis buffer. Cellular proteins were subjected to SDS-PAGE and western blot analysis with the following primary antibodies: anti-WW45 (Cell Signaling Technology), anti-Patched (Cell Signaling Technology), anti-FOXM1 (Abcam), anti-GAPDH (Santa Cruz), anti-Gli1 (Abcam), anti-ubiquitin (Cell Signaling Technology).

### GST pull-down assay

The coding sequence of WW45 was inserted into the expression vector pGEX-4T-1. The fusion protein GST-WW45 was purified. The whole cell lysates of A549 were prepared in 50mM Tris-Cl (pH7.5), 150mM NaCl, 0.1% NP40 and protease inhibitor cocktail. 5μg GST-WW45 fusion protein and 800μg cell lysates were incubated at 4°C for 3 hours. 20μl of glutathione-Sepharose-4B beads were added to the samples and incubated at 4°C for 1 hour to capture the GST fusion proteins. After washing with lysis buffer three times, the proteins were eluted in Laemmli buffer and analyzed by SDS-PAGE.

### Mass-spectrum assay

The HA-Gli1 plasmid was transfected into HEK293T cells. The immunoprecipitation was performed. The binding protein of Gli1 was separated by SDS-PAGE. The gel was stained with silver solution. The differential bands were collected and identified by mass spectrum.

### Luciferase assay

Cells were plated at a subconfluent density. 20 hours later, the reporter assays were performed using 0.1μg of GLIBS-Luc reporter construct, 0.5μg of expression vector, and 0.02μg of pRL-TK Renilla luciferase plasmid (internal control for transfection efficiency). Cell lysates were prepared 24 hours after transfection, and the reporter activity was measured using the dual-luciferase reporter assay system (Promega). Relative luciferase activity was calculated as the ratio of Firefly/Renilla luciferase activity.

### Ubiquitination assay

A549 cells were transfected with WW45 expression plasmid. 48 hours later, cells were treated with MG132 for 8 hours. Then cells were lysed and the immunoprecipitation was performed using anti-Gli1 antibody. The ubiquitination was examined using the anti-ubiquitin antibody.

### Immunofluorescence

24 hours after plating on cover slides, these cells were washed three times with ice-cold PBS and fixed in ice-cold methanol for 10 minutes. Cells were blocked with 3% BSA in PBST (PBS with 0.5% Tween-20) and then stained with primary antibodies diluted in 3% BSA, PBST overnight at 4°C. After washing four times in PBST, cells were incubated for 1 hour with the secondary antibody in 3% BSA, PBST at a 1:1000 dilution. Fluorescence was monitored by an inverted confocal laser microscopy.

### Construction of vectors

The coding sequence of WW45 was amplified by PCR and inserted into the expression vector pCMVTag2B using EcoRI and XhoI to obtain the Flag tagged WW45. Alternatively, the coding sequence of WW45 was amplified by PCR and inserted into the lenti-virus expression vector pCDH.

### Knocking down the expression of WW45

RNAi lenti-virus particles (sh-con, sh-WW45 and sh-Gli1) were from GeneChem (China). Cells were infected with the indicated lenti-virus particles of the same MOI for 24 hours and then stable knock-down cells were selected with the medium containing puromycin for at least a week.

### Crystal violet staining assay

Cells were seeded in 12-well plates with the medium containing 10% FBS. Medium was changed every other day. Ten days later, after removing the medium, the cells were fixed with 4% formalin and then stained with 0.5% crystal violet solution in 20% methanol. After staining for 20 min, the cells were washed twice with PBS and photographed. Cells were resolved with 1% SDS solution. The absorbance at 600nm was measured using a microplate reader.

### Boyden chamber assay

Cell migration was examined using Boyden chamber. Cells (2×10^5^) suspended in 50μl medium containing 1% FBS were placed in the upper chamber, and the lower chamber was loaded with 150μl medium containing 10% FBS acting as the chemoattractant. 6 hours later, cells migrated to the lower surface of filters was detected with traditional hematoxylin and eosin (H&E) staining. The experiments were repeated for thrice. Five random visual fields were counted for each sample and the average was determined.

### Transient transfection

Transfections were performed using Lipofectamine® 2000 (Invitrogene) as per the manufacturer's instructions.

### Xenograft assay

The control cells (A549/pCMVTag2B) and WW45 over-expressing A549 cells (A549/Flag-WW45) were injected into each flank of 4-week-old BALB/cA-nu/nu male nude mice. Four nude mice were used in this assay. Six weeks later, the mice were killed and the tumors were weighted and photographed.

### Mice and treatment

Mice were housed and treated after being approved by the Institutional Animal Care and Use Committee of Shanghai Chest Hospital, Shanghai Jiao Tong University. *Kras^G12D^* and *Lkb1^L/L^* mice were obtained from Jackson Lab (Koch Institute for Integrative Cancer Research, Cambridge, MA). Lung cancer mouse models with *Kras^G12D^*/*Lkb1^L^^/L^* mice were generated by crossing *Kras^G12D^* and *Lkb1^L/L^* mice. The coding sequences for WW45 and Cre were inserted into the pBabe vector. Each mouse was treated with 2×10^6^ PFU virus via nasal inhalation. The lung tissues were analyzed 12 weeks later after virus treatment.

### Histopathologic analysis

The lung tissues of the mice were isolated and fixed in 4% formalin. Lung lobes were embedded in paraffin, sectioned, and stained with H&E. Tumor number was counted under microscope.

### Statistical analysis

Statistical analyses were performed by the Student t-test (two-tailed) using GraphPad Prism software. Protein quantification was performed using ImageJ software. Differences with *P*<0.05 were considered statistically significant. Data were represented as mean±SEM.
